# Supplementary material for: Microglial Metamorphosis in Three Dimensions in Virus Limbic Encephalitis: An Unbiased Pictorial Representation Based on a Stereological Sampling Approach of Surveillant and Reactive Microglia
Source: Brain Sci. 2021 Jul 30;11(8):1009. doi: 10.3390/brainsci11081009 (PMC8393838; doi:10.3390/brainsci11081009)
Supplement: Supplementary file 1 [file brainsci-11-01009-s001.zip › brainsci-1262763-supplementary.pdf]

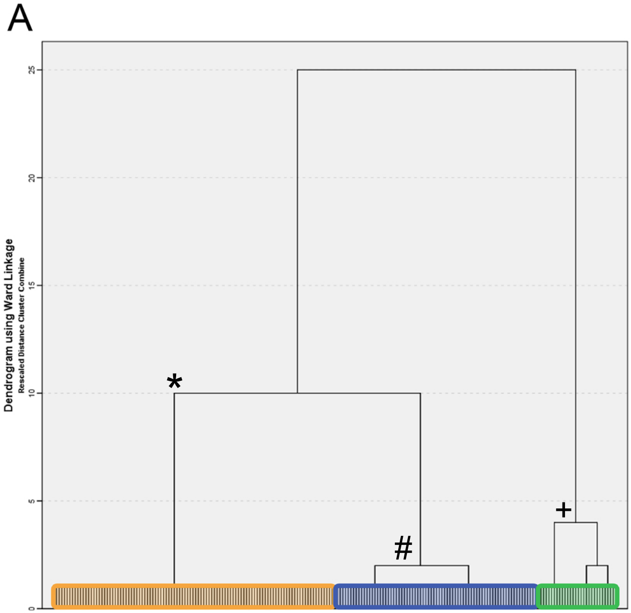

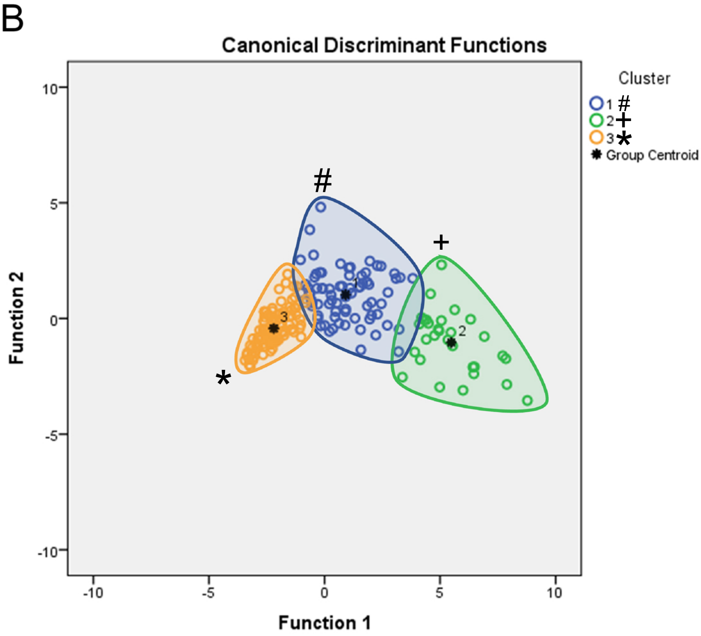

C

Eigenvalues

Function	Eigenvalue	% of Variance	Cumulative %	Canonical Correlation
1	7.091[a]	91.8	91.8	.936
2	.634[a]	8.2	100.0	.623

a. First 2 canonical discriminant functions were used in the analysis.

D

Wilks' Lambda

Test of Function(s)	Wilks' Lambda	Chi-square	df	Sig.
1 through 2	.076	531.826	10	.000
2	.612	101.124	4	.000

E

Structure Matrix

	Function	
	1	2
Convex Hull Surface (μm^2)	.861*	.327
Total Branch Volume (μm^3)	.685*	.193
Complexity	.507*	-.313
Mean Branch Volume (μm^3)	.332	.446*
Tortuosity	.232	.429*

Pooled within-groups correlations between discriminating variables and standardized canonical discriminant functions
Variables ordered by absolute size of correlation within function.

*. Largest absolute correlation between each variable and any discriminant function

F

Classification Results[a]

		Cluster	Predicted Group Membership			Total
			1	2	3	
Original	Count	1	72	2	1	75
		2	0	30	0	30
		3	2	0	104	106
	%	1	96.0	2.7	1.3	100.0
		2	.0	100.0	.0	100.0
		3	1.9	.0	98.1	100.0

a. 97.6% of original grouped cases correctly classified.
